# Concrete Made with Partially Substitutions of Copper Slag (CPS): A State Art of Review

**DOI:** 10.3390/ma15155196

**Published:** 2022-07-27

**Authors:** Jawad Ahmad, Ali Majdi, Ahmed Farouk Deifalla, Haytham F. Isleem, Cut Rahmawati

**Affiliations:** 1 Department of Civil Engineering, Military College of Engineering, Risalpur, Sub Campus of Natioanl University of Sciences and Technology, Islamabad 44000, Pakistan; 2 Department of Building and Construction Technologies and Engineering, Al-Mustaqbal University College, Hillah 51001, Iraq; alimajdi@mustaqbal-college.edu.iq; 3 Structural Engineering Department, Faculty of Engineering and Technology, Future University in Egypt, New Cairo 11845, Egypt; 4 Department of Construction Management, Qujing Normal University, Qujing 655011, China; hathamisleem@mail.qjnu.edu.cn; 5 Department of Civil Engineering, Universitas Abulyatama, Aceh Besar 23372, Indonesia; cutrahmawati@abulyatama.ac.id

**Keywords:** copper slag, mechanical strength, flowability, chemical composition and microstructure analysis

## Abstract

Copper slag (CPS) is a large amount of waste material produced during the manufacture of copper. The disposal of this waste material becomes a problem for environmental concerns. Therefore, it is necessary to explore feasible alternate disposal options. They may also be utilized in concrete manufacturing to cut down on the usage of cement and natural aggregates. A lot of researchers focus on utilizing CPS in concrete, either as a cement replacement or as a filler material. This article aims to summarize the literature already carried out on CPS in conventional concrete to identify the influence of CPS on the fresh, hardened and durability performance of cement concrete. Results indicate that CPS improved the strength and durability performance of concrete but simultaneously decreased the slump value of concrete. Furthermore, an increase in the durability performance of concrete was also observed with CPS. However, the higher dose results declined in mechanical and durability aspects owing to a scarcity of flowability. Therefore, it is suggested to use the optimum dose of CPS. However, a different researcher recommends a different optimum dose ranging from 50 to 60% by weight of fine aggregate depending on the source of CPS. The review also recommends future researcher guidelines on CPS in concrete.

## 1. Introduction

A widely utilized raw material in construction is concrete, which is the basis for all construction and development initiatives around the globe, serving as the base for all buildings and infrastructure [1,2,3]. The environmental impact of concrete’s primary ingredients changes depending on the kind of concrete and the amount of cement applied. Because concrete is utilized in such huge amounts across the globe, it raises several questions about its long-term viability [4]. An increase in the amount of riverbed sand and gravel, which are used as concrete components, is raising significant worry among environmentalists. The increased removal of natural sand from riverbeds has come from the extensive usage of concrete, which has occurred because of the rapid urbanization and industrialization of the world’s population. Enhancement of riverbed distance, a decrease in the water table, revelation of bridge substructures, the most significant influence on rivers, deltas, coastal and marine ecologies, land loss as a result of the river or coastal erosion and a reduction in the quantity of deposit sources are just a few of the negative consequences of sedimentation [5]. Moreover, owing to limits on removal of sand from rivers, the construction industry’s viability has been seriously threatened, resulting in a significant increase in sand charges [6,7]. Fine aggregates in concrete may be made from a variety of industrial wastes [8,9,10].

The term “sustainable building” refers to management that is accountable for providing a favorable environment that considers ecological and resource development [9,10,11,12,13]. Concrete is rapidly becoming a crucial building materials across the globe, due to its low superior performance. However, manufacturing cement has an impact on ecological systems [14,15,16]. Cement production, which is a major constituent in concrete, is a considerable source of greenhouse gas discharges CO_2_ [17,18,19]. Currently, the globe generates about 3.6 billion metric tons of material every year [20]. The amount is predicted to reach more than 5 billion metric tons by 2030 [17,21]. Despite the fact that each country’s situation varies, over half of the world’s ordinary Portland cement (OPC) generates 11 billion metric tons of concrete each year, with the balance being utilized in projects [22]. To minimize CO_2_ emissions, waste materials should be used instead of cement in concrete.

The industrial sector has seen significant expansion, resulting in a vast number of by-products whose dumping has come to be a serious problem, since it impacts the ecosystem’s [23,24,25]. The use of such relevant by-products in the building sector, particularly in concrete manufacturing, will help to reduce environmental stress. Several studies have already demonstrated that using industrial waste such as fly ash [25], rice husk ash [26], bagasse ash [27], silica fume [28], blast furnace slag [29], copper slag [30], waste glass [31] and waste marble [32], etc., were considered to be advantageous. Similar copper slags are also valuable options to be used as concrete ingredients.

Copper slag (CPS) is a metallurgical waste product that is created through the matte smelting of copper metal. CPS is an industrial waste substance that is formed as a by-product of the copper production process. It is a smooth and glassy by-product of the matte smelting and refining phases involved in the pyrometallurgical removal of copper. Copper is extracted and purified from copper oxide ores using aqueous (water-based) solutions at room temperature, often in three steps: heap leaching, solvent extraction and electrowinning.

**Figure 1:**
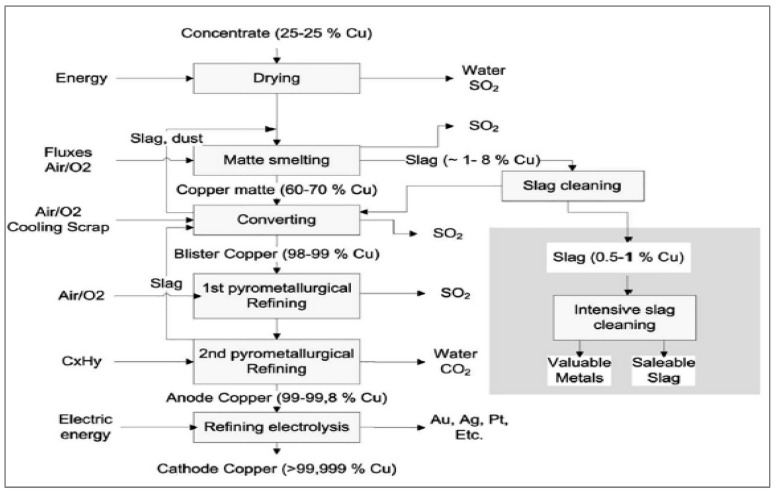
Flow Chart of CPS Extraction [33].

**Figure 2:**
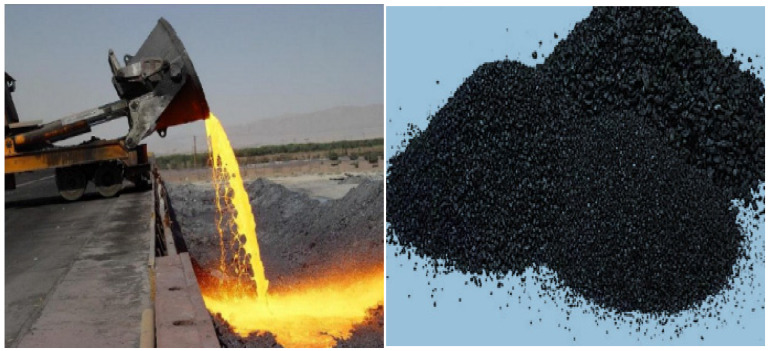
Cooling of CPS in air [33,34].

1. Heap leaching is the method of extracting metals from chemical solutions by allowing them to percolate. Low-grade ore that would otherwise not be economically sent through a milling process is often utilized in heap leaching. The crushed ore is placed into a heap on top of an impermeable layer, on a little slope, after mining, shipping and crushing to a constant gravel or golf ball size. The copper from the ore is dispersed in the leaching agent (diluted sulfuric acid), which is sprayed via sprinklers on top of the heap pile and allowed to flow down into the heap. A small pool is used to collect the copper sulfate and sulfuric acid “pregnant” leach solution that results. Currently, concentrations of the copper complex range from 60 to 70 percent.
2. The second stage is solvent extraction, which involves stirring and allowing two immiscible (non-mixing) liquids to separate, causing the copper to transfer from one liquid to the other. A solvent is aggressively combined with the pregnant leach solution. The copper migrates into the solvent from the leach solution. The two liquids are then allowed to separate depending on solubility, with the contaminants staying in the leach solution while copper remain in solution in the solvent. The remaining leach solution is then recycled by adding more acid and returning it to the heap leaching sprinklers.
3. The last stage is an electrolysis process known as electrowinning. An inert anode (positive electrode) and the copper solution from the prior phase, which functions as an electrolyte, are both contacted by an electrical current. Next, 99.99 percent pure copper is deposited onto a cathode (negative electrode) as positively charged copper ions (referred to as cations) emerge from solution. The manufacture process of copper slag in the industry is displayed in Figure 1. CPS is often a dark black color, as seen in Figure 2.

It has been calculated that the copper industry generates about 24.6 million tons of slag per year across the globe. Even though CPS is frequently employed in the sandblasting business and the manufacture of abrasive tools, the rest is placed without any further usage or recovery possible. CPS contains mechanical and chemical properties that allow it to be utilized in concrete as a substitute for cement or sand, depending on the application. For example, copper slag possesses a variety of mechanical features that make it a desirable choice for aggregate application, including great soundness characteristics, outstanding abrasion resistance and excellent stability [35]. Because it takes 2.2 million tons of CPS to generate each year [35], environmental protection organizations and governments are concerned about the usage and disposal of this CPS waste. 

Following the escalation of the issue, various investigations have shown a wide range of potential reuse and recycling options for this particular material. Among the potential options for metal recovery from slags containing significant levels of metallic elements are different techniques such as electric arc furnace melting, leaching and flotation (as well as other methods). However, since the metals present in copper slag are often found in trace levels, their recovery may not be economically feasible in most cases. Instead, various applications for copper slag were investigated and eventually accepted [35]. An alternate conceivable use for this material might be as an aggregate and perhaps as a cement substitute in the manufacturing of concrete, based on its physical and chemical qualities. 

Additionally, copper slag has pozzolanic capabilities due to its low CaO level, as well as the presence of other oxides such as Al_2_O_3_, SiO_2_, and Fe_2_O_3_. Pozzolans are classified as siliceous or siliceous and aluminous minerals that have no or little cementitious properties; however, when finely ground, they chemically react with calcium hydroxide (CH) in the presence of water to produce compounds with cementitious characteristics (calcium silicate hydrates). It is possible to reduce disposal costs by using CPS in concrete as binder or filler. This may also assist with conserving the environment by lowering the amount of waste produced. Because of the massive amount of CPS being generated, environmental pollution has an adverse effect on the growth of the nation. The effective and environmentally friendly use of CPS, as well as the promotion of green building, are significant concerns of this assessment.

Although some studies have discovered that copper slag possesses pozzolanic activity, the activity is quite low, limiting the use of copper slag as a mineral additive in concrete [36,37].

The silica modulus (ratio of the actual amount of lime in raw meal/clinker to the theoretical lime required by the major oxides (SiO_2_, Al_2_O_3_ and Fe_2_O_3_) in the clinker) of the activator increases the hydration degree of quick-cooled copper slag and the polymerization of amorphous hydration products. In quickly cooled copper slag, instead of high Fe particles, high Si particles provide primary activities. Alkali-activated quick cooled copper slag mortars exhibit reasonably high compressive strengths, particularly when the silica modulus is greater than one, indicating that alkali-activated copper slag for building may be feasible in the future [38]. As the silica modulus decreases, the intensity of the reaction rises. However, silica modulus reductions have a detrimental effect on the mechanical characteristics of the resulting concrete. The ideal silica modulus requirement is determined by the raw sample’s mineralogical makeup. The silica modulus of the used alkaline solution determines the rate of partial dissolution of the semi-crystalline and crystalline mineral phases found in natural pozzolan [39].

The dissolution of soluble components in raw materials, the accumulation of soluble components and the creation of oligomers, and the polymerization of oligomers and the precipitation of hydration products are the three steps of the early hydration process of alkali-activated material [40]. Some active components in copper slag, such as Si, Al and Ca, are thought to dilute when exposed to high OH- concentrations. The concentration of ions then rises, and oligomers form. The hydration products then precipitate, linking the unreacted copper slag particles [41]. A study [42] concluded that the microstructure is densified by adding CPS up to 60% because of its pozzolanic action.

## 2. Physical and Chemical Compositions of CPS

It is possible to determine the applicability and ability of using industrial wastes in concrete based on their physical properties, which include specific gravity, absorption coefficients, grain size, fineness modulus, moisture content, bulk density specific surface area and unit weight. According to previous investigations, the physical parameters of CPS are listed in Table 1. CPS has specific gravity ranging from 2.4 to 3.5, which is somewhat higher than the specific gravity of aggregate (2.4 to 3.5). CPS has an absorption capability of 0.36 percent according to published data, as shown in Table 1. Because the absorption capacity of CPS is lower than that of fine aggregate, the flowability of concrete will increase. It is composed mostly of particles with uniform, angular shapes, with the majority of the particles measuring between 4.75 and 0.075 mm in size. [43]. However, a study suggested that the particle size of copper slag should be below 10 mm [44]. Copper slag has a density ranging between 3.16 and 3.87 kg/m^3^, which fluctuates depending on the quantity of iron included in it.

**Figure 3:**
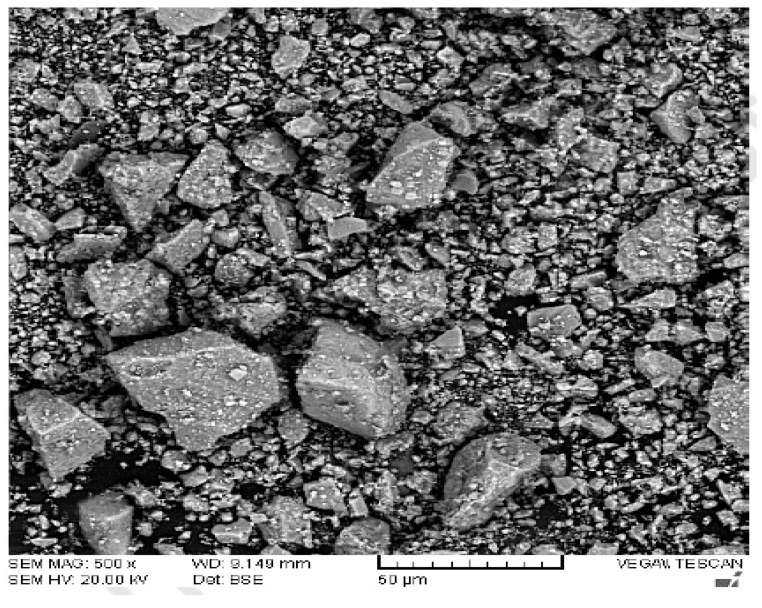
SEM of CPS [49].

The scan electronic microscopy (SEM) of CPS is shown in Figure 3. The particles have an uneven morphology, rough and irregular in shape, as may be expected. Furthermore, it is obvious that CPS has a reasonably smooth surface, which is responsible for the greater workability of new concrete using CPS as a partial fine aggregate when compared to a mix made with 100 percent natural sand [48].

Four to five percent alumina, four to six percent calcium oxide, thirty-five percent to thirty-seven percent iron, thirty percent to thirty-four percent silica, and one percent copper are the primary elements of CPS [50]. Other slag aggregates, such as electric furnace ferronickel slags, have a chemical composition that is predominantly composed of SiO_2_, MgO and Fe_2_O_3_ as the primary constituents [51]. Iron slag is mostly composed of the elements SiO_2_, Al_2_O_3_, CaO and MgO, which account for 95 percent of the total composition. It also contains manganese, iron and sulfur compounds, as well as tiny quantities of numerous other elements [52]. According to previous research, the chemical makeup of CPS is shown in Table 2.

**Figure 4:**
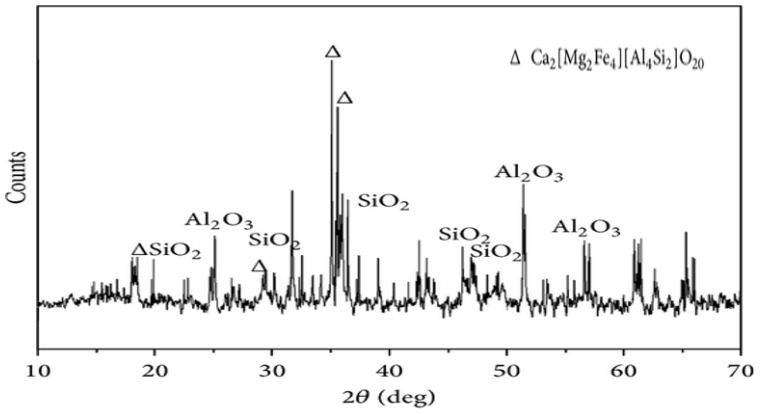
XRD test results of CPS [58]: Used as per Elsevier permission.

X-ray diffraction (XRD) analysis revealed that the mineralogical components included in this slag are pyroxene (CaZnSi_2_O_6_), fayalite S(iO_4_Fe_2_), anorthite (CaAl_2_Si_2_O_8_), quartz (SiO_2_) and magnetite (Fe_3_O_4_) [53]. Figure 4 shows the results of the XRD analysis (Fe_3_O_4_). The amorphous nature of the SiO_2_ found in CPS has a significant impact on concrete, from the initial hydration to the ultimate strength [56]. According to ASTM [57], it is possible to employ pozzolanic materials that have accumulated more than 70% of a chemical (silica dioxide, calcium oxide, aluminum oxide, magnesium oxide, sodium oxide and iron oxide). From the Table 2, CPS has a greater than 70% accumulation of the mentioned chemical, making it suitable for use as a cementitious material.

## 3. Fresh Properties

### 3.1. Workability of Concrete 

**Figure 5:**
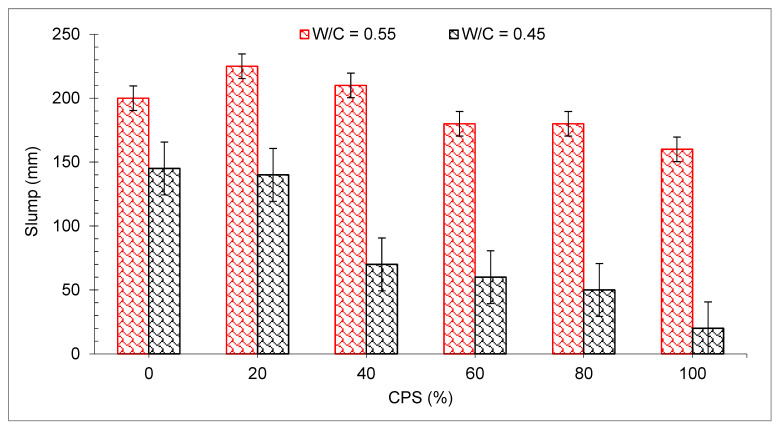
Workability of concrete [47].

Figure 5 shows the outcomes of the flowability of concrete for each CPS proportion ratio for each of the two distinct waters to binder ratios (*w*/*c*). Concrete made with *w*/*c* 0.55 possess high flowability (100–175 mm) to have a maximum slump value of 175 mm, and therefore higher workable concrete. However, the slumps of copper slag mixes were less than control concrete for a 0.45 *w*/*c* and high percentages of copper slag are substituted. In such a case, concrete has very little workability, which could be unsatisfactory for several useful purposes. Furthermore, lower workability also adversely affects strength properties.

**Figure 6:**
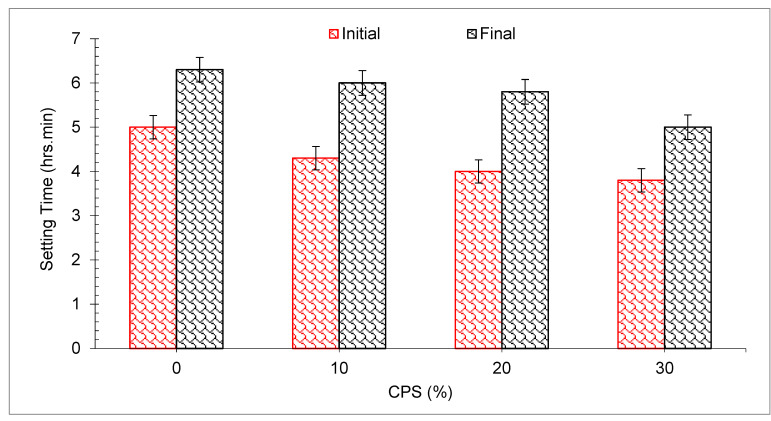
Setting time of mortar with CPS [64].

It was hypothesized that the CPS would experience more slump collapse (more workable concrete) when compared with natural aggregate due to lower water absorption. However, the findings proved that the supposition is wrong. Slumps were probably influenced to some degree due to the angular shape of CPS, which enhances the friction between concrete components and ultimately reduced the flowability of concrete. In addition, a researcher discovered that when sand was substituted with CPS, the workability of the concrete decreased, contrary to what was expected [59]. Workable mixtures may be created for all sand substitute ratios provided the water is properly managed. CPS particles have a glassy and smooth surface, which might be a contributing factor to the enhanced slump value of concrete mixtures [49]. Furthermore, the SEM picture (Figure 3) of CS revealed that CPS is composed mostly of spherical particles which allow for more efficient movement of ingredients. There was also a modest delay in the setting time mixtures when CPS was added to the mix, as shown in Figure 6, which might be related to the existence of heavy metals in the CPS that delayed the hydration of the cement during the setting process [60]. 

According to one investigation, the recorded slump was 150 mm when CPS was utilized to substitute 100 percent of the copper. According to the authors, the low water absorption features of CPS and its glassy surface when compared to aggregate caused in a noteworthy increase in the flowability of concrete due to a significant rise in the amount of free water remaining after the absorption and hydration processes were completed. The improvement in flowability of concrete may be made with the same amount of sand substituted, and these mixes may have better flowability, as well as higher strength and durability than standard high-performance concrete (HPC) mixes. Furthermore, it was highlighted that mixes containing significant levels of CPS exhibit bleeding and segregation which might have a harmful influence on the strength of concrete [61].

### 3.2. Setting Times of Mortar

Figure 6 depicts the start and final setting times for the cement that will be blended with different percentages of CPS. It can be noted that the initial and final setting time of 10 percent substation of CPS is much less than the cement. It is well known that CPS reacting passively with cement hydrates results in the creation of calcium silicate hydrates CSH gel and calcium sulfate, and this reaction helps to construct the structure of cement pastes during the hydration process and hence shortening the setting time of the cement. As the percentage of CPS replacement is increased to 20 percent and 30 percent, reduced setting times are observed when compared with the percentages of 0 and 10 percent of cement paste mixed with CPS, respectively, because of the larger need for water with a higher concentration of CS, which results in a denser structure and hence, a shorter setting time. In addition, according to one research study, slag will aid in the setting of cement paste by lowering the induction time [62]. With the smaller particles and greater slag dose, the initial setting time was reduced, and the induction time was shortened. However, because of the reduction in the particle size of CPS, the research discovered a much greater delay in the setting time [63]. The delay in setting time with CPS substitution may be due to pozzolanic reaction, as the pozzolanic reaction continues gradually, as associated with hydration of cement.

## 4. Mechanical Strength

### 4.1. Compressive Strength (CMS) of Concrete

**Figure 7:**
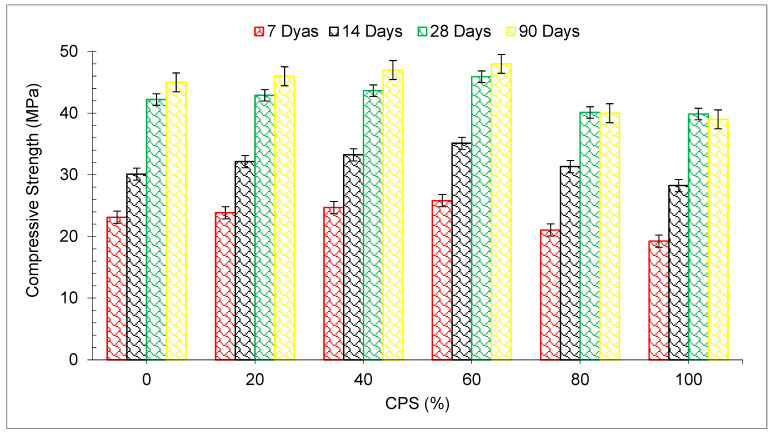
Compressive strength of concrete [45].

The compressive strength (CMS) outcomes at each curing interval are shown in Figure 7, with variable percentages of CPS substation instead of natural sand. It is seen that as the proportion of CPS replacement ratio by weight of river sand rises, in an increase in CMPS is seen up to 60% substitute of aggregate with CPS. However, a minor drop in CMS is seen for the mixes containing 80 and 100 percent CPS, respectively, when compared with the blank mix (control mix). The maximum CMS at 7, 14, 28 and 90 days curing was 11.64 percent, 16.60 percent, 6.89 percent, and 9.66 percent in comparison with blank concrete at 7, 14, 28, and 90 days, respectively. The use of small particles of CPS in concrete improves the packing density and makes it less permeable. Additionally, CPS particles have angular edges which aid in the enhancement of matrix cohesion by increasing the surface area of the particle [54]. Additionally, according to research, the use of sharp-edged waste copper slag particles increases the bonding of concrete with its other component elements. As a result, improvement in strength is seen until the mix contains 60 percent CPS. Through compressive strength tests, it has been shown that CPS may be partially substituted with fine aggregate up to 60% for M40 grade concrete without impacting the strength qualities of concrete [65]. The CMS of the mortar improves gradually when the curing period is increased with the addition of mineral additives. When slag is used in place of cement, the concentration of the hydration component Ca(OH)_2_ decreases, while small particles of mineral mixture fill the spaces between cement particles, making the cement mortar denser and strengthening the interfacial area [66]. According to the findings of the study, the CMS of concrete is equivalent to the control concrete up to 40 percent substitution of CPS [33]. Under sulfate exposure circumstances, again, mass is found in CPS concrete, causing in a reduction in CMS [67]. The majority of the studies have observed a continuous rise in CMS, with an enhancement in CPS content up to a 50 percent proportion of sand [61,67]. However, the optimum dose of CPS varies due to different sources of CPS. Table 3 displays the summary of CMS with partial substitution of CPS in concrete, as per past studies.

**Figure 8:**
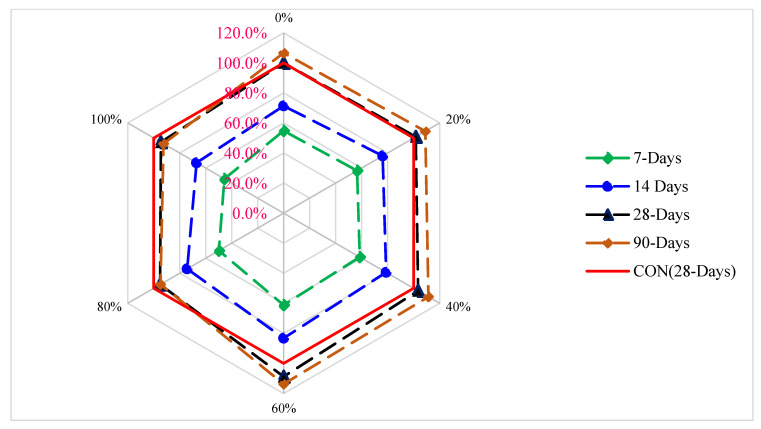
Relative compressive strength of concrete: data source [45].

Figure 8 displays the strength age relation of CMS with different proportions of CPS at various periods of curing. CMS (28 days) of the blank mix (control) is chosen as benchmark strength (reference strength). From Figure 8, the maximum CMS is achieved at 60% substitution of CPS. CMS at 7 days and 14 days is 39% and 17% lower than reference compressive strength at 60% substitution of CPS. However, CMS of concrete at 28 and 90 days is 9% and 14% greater than the reference CMS at 60% substation of CPS. It can be concluded that the CPS up to 60% can be used in concrete without any harmful effect on the compressive strength.

### 4.2. Split Tensile Strength (TS)

**Figure 9:**
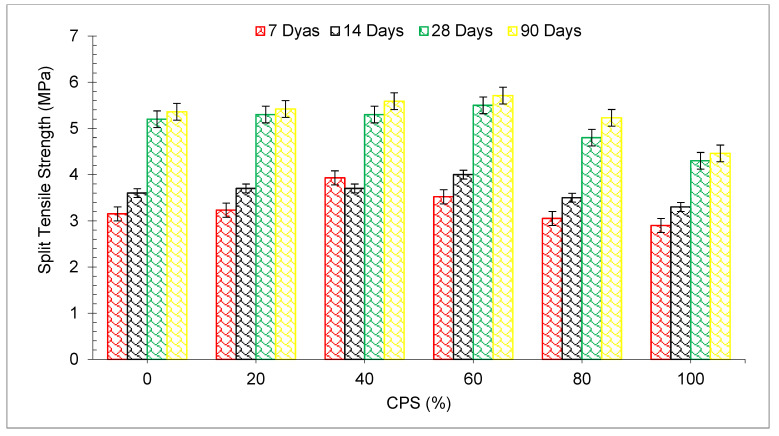
Split tensile strength of concrete [45].

Figure 9 represents the split tensile strength (TS) test results of all mixes (0 to 100 CPS substitution) at each curing period, respectively. In the same way, as the partial replacement of CPS by river sand grows, the TS improves as the partial replacement of CPS by river sand increases, up to a maximum of 60 percent substitution of CPS instead of natural river sand in comparison to mixing. Following that, a minor drop in TS is seen for the mixes with 80 percent and 100 percent replacement of CPS, respectively as compared to the control mix. When compared to the control mix, the 60 percent substation of CPS achieved maximum TS of 11.75 percent, 11.05 percent, 4.69 percent and 6.53 percent at 7-, 14-, 28-, and 90-day curing periods, respectively, compared to the control concrete TS. The filling effects of tiny particles of discarded CPS are responsible for the improvement in TS. Furthermore, according to one research study, the compressive, tensile and flexural strengths of concrete were equivalent to those of the control mix when up to 50% CPS replacement by weight of sand was used, but the TS started to drop when the CPS concentration increased further, up to 80% [54,63,68,69]. 

Numerous studies have indicated that the CMS and TS of concrete specimens created using CPS as fine and coarse particles are higher than those of regular concrete [61]. As a consequence of using CPS aggregate rather than natural aggregate, the CMS of the concrete improved by about 10–15 percent after 28 days and the TS improved by approximately 10–18 percent after 28 days [61]. The addition of CPS enhanced the tensile property of the TS by up to 40 percent replacement. Following that, it gradually decreased, but did not fall below the maximum TS obtained with fly ash [70]. In most cases, the use of CPS as a partial replacement in concrete produced with 100 percent cement resulted in an increase in TS, except for concrete containing 100 percent CPS [49]. According to the findings of one investigation, concrete made with CPS as a partial substitute had greater TS up to 60%. A further increase in the usage of CPS over this replacement ratio resulted in a 7–10 percent decrease in the TS [71]. Furthermore, Table 3 displays the summary of TS of concrete with various percentages of CPS.

**Figure 10:**
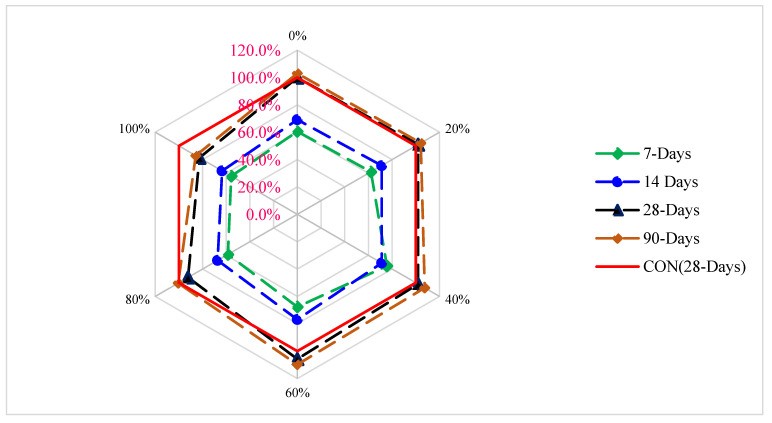
Relative split tensile strength of concrete: data source [45].

Figure 10 shows the TS age relation with distinct percentages of CPS at various periods of curing. TS (28 days) of the blank mix (control) is taken as a reference mix. It can be noted that the maximum TS is achieved at 60% substitution of CPS instead of natural river sand. TS at 7 days and 14 days is 33% and 23% lower than reference TS at 60% replacement of CPS instead of natural river sand. However, TS of concrete at 28 and 90 days of curing is 6% and 10% more than the reference TS at 60% substation of CPS. However, Wang et al. reported a 40% optimum dose [44]. At a higher dose (80 and 100%), split tensile is lower than the reference TS, even at 90 days of curing. 

**Figure 11:**
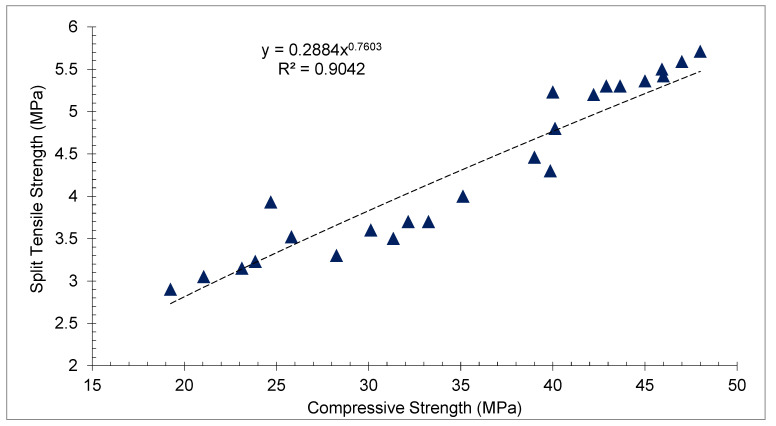
Correlation between CMS and TS: data source [45].

Figure 11 shows the correlation between compressive strength (CMS) and split tensile strength (TS) with various proportions of CPS at different days of curing. TS depends on the CMS of concrete. TS is about 10 to 50% of CMS of concrete. It can be noted that the trendline between CMS and TS seems to be straight. Therefore, a strong correlation exists between CMS and TS with an R^2^ value greater than 90%. 

### 4.3. Flexural Strength (FS)

**Figure 12:**
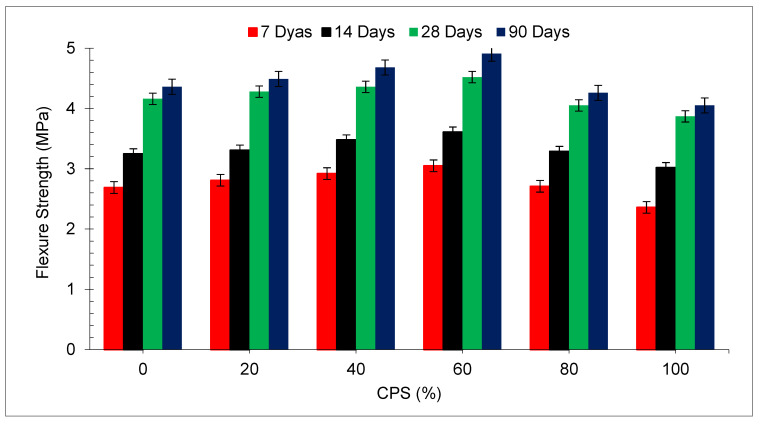
Flexural strength of concrete: data source [45].

The flexural strength (FS) of concrete for all mixtures at each curing time is shown in Figure 12. It can be shown that as the percentage of CPS replaced by river sand rose, an increase in FS was noted up to a mix of 60% proportion of CPS when compared with a mixture of 0% CPS (control). The CMS and TS tests both indicated an increasing trend in progress over time. Further increases in the substitution ratio of CPS resulted in a minor reduction in FS for the mix, and 80 and 100% substation of CPS when compared with the control mix. Comparing the FS of the mix (60% substitution of CPS) to the control mix, it can be shown that the maximum FS of the mix (60% substitution of CPS) was 13.38 percent, 11.08 percent, 8.65 percent and 12.61 percent after 7, 14, 28 and 90 days of curing time, respectively. The use of tiny particles of CPS improves the interlocking ability of component materials, which results in an increase in FS and stiffness of concrete [72]. 

Analysis of FS of the mortar containing 10 percent CPS reveals that FS increased by 12.9 percent more than the control mixture. According to some theories, this improvement may be attributed to the increase in the compactness and durability matrix of the CPS mortar. Despite this, the findings of the 20 and 30 percent CPS tests indicate that the gain in FS is much more significant. The improvement in FS of the 20 percent CS mortar was about 18.2 percent higher than the FS of the control mortar. The concrete containing 30 percent CPS resulted in the greatest improvement in FS, which was 38.7 percent more than the control mortar. This substantial increase in FS was most likely due to the increased compaction and uniformity of the distribution of CPS in mortar mixes, which has resulted in a successful improvement in FS [73]. The FS of concrete was equivalent to that of the control mix when up to 50 percent CPS replacement for sand was used, but it declined when the CPS content of the concrete increased further [54]. The FS of concrete was improved when 40 percent CPS was used, but the FS reduced when the amount of CPS used exceeded 40 percent. The lowest FS of 6.16 MPa was achieved when 100 percent CPS was used, and this was achieved by adding 1 percent nano silica to the 100 percent copper slag mix. The FS of concrete improved from 6.16 to 6.49 MPa throughout the testing process [74]. Furthermore, Table 3 displays the summary FS of concrete with different percentages of CPS.

**Figure 13:**
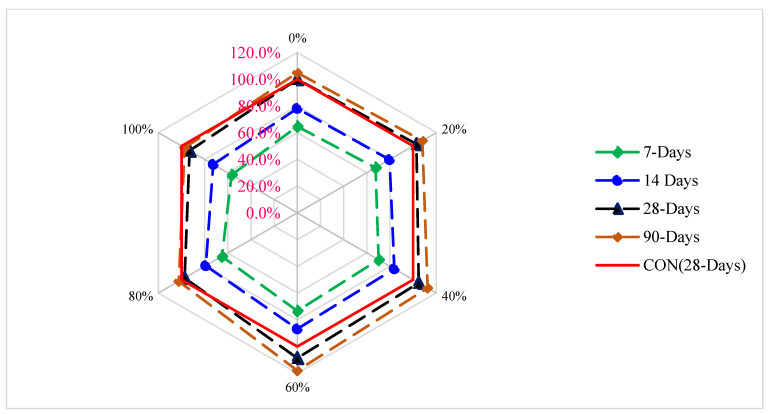
Relative flexural strength of concrete: data source [45].

Figure 13 reveals the relative flexural strength (FS) of concrete with various percentages of CPS at different periods of curing. FS (28 days) of the blank mix (control) is taken as the reference mix. It can be noted that maximum flexural strength is attained at 60% replacement of CPS instead of natural river sand. FS at 7 days and 14 days is 36% and 13% lower than reference FS at 60% substitution of CPS instead of natural river sand. However, the FS of concrete at 28 and 90 days is 9% and 18% higher than the reference FS at 60% substation of CPS. At a higher dose (80 and 100%), FS is lower than the reference tensile strength even at 90 days. Therefore, it is recommended that CPS is used up to 60% substation instead of the natural river without any negative effect on the flexural strength of concrete.

**Figure 14:**
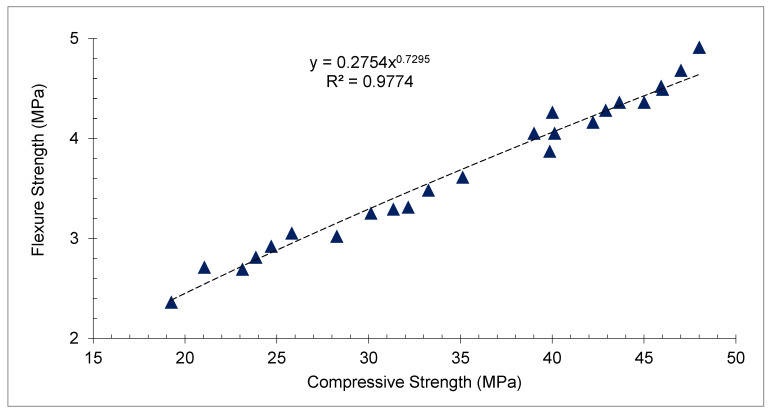
Correlation between compressive and flexural strength: data source [45].

Figure 14 reveals the correlation between the compressive and flexural capacity of concrete with distinct percentages of CPS at various days of curing. *FS* is almost 10% to 20% of *CMS* varying on the mix design of concrete. It can be noticed that the trendline among CMS and FS seems to be straight. Therefore, a strong correlation has occurred among CMS and FS with an R^2^ value more than 90%. 

**Table 3:** Summary of slump and mechanical strength of concrete with copper slag (CPS).

Reference	Percentage of	Slump	Compression												Flexural						Split Tensile											
	(CPS)	(mm)	Strength (MPa)												Strength (MPa)						Strength (MPa)											
[47]	W/C=0.55		7D						28D						-						7D						28D					
	0%	200	23.9						34.6												2.4						2.7					
	20%	225	29.0						38.6												2.5						2.8					
	40%	210	25.7						33.2												2.3						2.7					
	60%	180	25.8						34.0												2.4						2.8					
	80%	180	24.5						32.8												2.3						2.6					
	100%	160	23.6						30.7												-						-					
[30]		-	7D			28D			56D			90D			28D						28D											
	0%		30			44			43			45			7						3.5											
	20%		34			45			45			47			7						3.8											
	40%		35			47			50			53			7.5						4											
	60%		33			45			47			50			7						4											
	80%		33			46			48			51			6.5						3.9											
	100%		34			46			46			50			6.5						3.9											
[53]		-	7D				28D				90D				28D			90D			28D						90D					
	0%		29.7				40.0				44.0				3.28			3.35			3.74						4.05					
	5%		27.5				37.5				43.1				3.09			3.30			3.72						4.02					
	10%		25.2				36.0				41.7				3.02			3.17			3.71						4.02					
	15%		23.5				35.2				39.5				2.98			3.12			3.67						3.98					
[54]	0%CS	65	7D			28D			56D			90D			28D						28D											
	10%CPS+90%S	80	23.3			24.6			25.3			27			7.7						3											
	20%CPS+80%S	80	29			31			34.7			36			7.2						3.5											
	40%CPS+60%S	110	30.6			39.8			40			42			6.5						3.8											
	50%CPS+50%S	130	30			42.7			44.5			50.3			7.3						4.1											
	60%CPS+40%S	165	28			39.2			42			47.8			6.3						3.6											
	80%CPS+20%S	190	26.8			35			40.1			44.8			7.2						3.6											
	100%CPS	200	23.3			26.1			32			35.5			5.9						3.4											
[45]			7D			14D			28D			90D			14D		28D		90D		14D				28D				90D			
	0%	65	23.12			30.12			42.21			45			3.25		4.16		4.36		3.6				5.3				5.36			
	20%	70	23.85			32.15			42.90			46			3.31		4.28		4.49		3.7				5.3				5.42			
	40%	72	24.69			33.26			43.65			47			3.48		4.36		4.68		3.7				5.3				5.59			
	60%	75	25.81			35.12			45.92			48			3.61		4.52		4.91		4.0				5.5				5.71			
	80%	82	21.05			31.34			40.12			40			3.29		4.05		4.26		3.5				4.8				5.23			
	100%	80	19.25			28.26			39.86			39			3.02		3.87		4.05		3.3				4.3				4.69			
[61]			7D						28D						28D						28D											
	0%CS	28	76.9						93.9						14.6						5.4											
	10%CPS+90%S	28	79.6						99.8						13						5.2											
	20%CPS+80%S	50	74.5						95.3						12.4						6.2											
	40%CPS+60%S	85	76.4						95.2						12.5						6.1											
	50%CPS+50%S	115	77.8						96.8						12.9						6.1											
	60%CPS+40%S	128	69.0						83.0						11.1						4.8											
	80%CPS+20%S	143	63.8						83.6						10.3						4.7											
	100%CPS	150	63.4						82.0						10.1						4.4											
[49]			14D						28D						-						14D						28D					
	0%	52	32.1						35.7												3.77						3.9					
	25%	57	37.1						38.5												4.3						4.36					
	50%	63	38.2						39.9												4.37						4.43					
	75%	68	31.8						34.1												4.24						4.29					
	100%	74	28.8						30.0												4.13						4.18					
[74]		-	28D												28D						-											
	0%		93												6.2																	
	20%		97												6.5																	
	40%		100												7.1																	
	60%		95												6.9																	
	80%		91												6.4																	
	100%		87												6.1																	
[75]		-	7D						28D						7D			28D			7D						28D					
	0%		35						40						4.0			5.0			2.5						3.0					
	5%		32						43						4.1			5.1			2.6						3.3					
	10%		30						41						4.0			5.0			2.5						3.2					
	15%		28						32						3.2			4.0			1.7						2.5					
[76]		-	28D						56D						28D			56D			28D						56D					
	0%		45						49						3.6			4.0			3.6						4.0					
	5%		50						55						3.5			4.5			3.5						4.5					
	10%		47						51						3.3			4.4			3.3						4.4					
	15%		45						49						3.1			4.2			3.1						4.2					
	20%		40						47						3.0			4.0			3.0						4.0					
	25%		38						44						2.9			3.9			2.9						3.9					
	30%		36						42						2.6			3.6			2.6						3.6					
[77]		-	7D				14D				28D				-						7D				14D				28D			
	0%		17.03				21.66				29.25										1.82				2.03				2.73			
	10%		18.74				23.70				29.85										2.12				2.19				2.95			
	20%		20.22				25.22				32.07										2.21				2.31				3.09			
	30%		23.11				27.33				37.55										2.22				2.38				3.42			
	40%		24.66				28.59				39.48										2.38				2.5				3.49			
	50%		20.96				25.9				33.03										2.05				2.26				2.48			
	60%		16.48				20.45				28.66										1.98				2.12				2.33			
[65]		-	28D						56D						-						28D						56D					
	0%		30						31												10.66						11.72					
	20%		35						37												9.94						10.59					
	40%		36						38												10.81						10.53					
	60%		39						38												10.43						11.14					
	80%		42						42												11.07						11.70					
	100%		36						36												12.18						1257					
[33]		-	7D						28D						-						7D						28D					
	0%		24						33												2.8						3.3					
	20%		26						28												3.0						3.4					
	40%		31						37												3.3						3.5					
	60%		26						31												3.0						3.2					
	80%		25						28												2.9						3.1					
	100%		20						21												2.5						2.6					
[78]			28D												-						-											
	0%	29	29.19																													
	10%	34	31.56																													
	20%	43	34.59																													
	30%	46	41.7																													
	40%	51	38.74																													
	50%	55	42.22																													
	60%	57	34.81																													
	70%	62	32.74																													
	80%	66	31.7																													
	90%	69	30.15																													
	100%	78	30																													
[64]		-	7D						28D						7D			28D			-											
	0%		40						45						8.0			9.0														
	10%		42						47						10.0			10.0														
	20%		43						49						11.0			10.0														
	30%		45						50						11.5			12.5														
[79]		-	7D						28D						28D																	
	0%		42						62						3.5						-											
	20%		50						62						3.4																	
	40%		52						70						3.5																	
	60%		50						68						3.3																	
	80%		40						60						3.2																	
	100%		30						50						2																	
[80]		-	7D			14D			28D			56D			28D						7D			14D			28D			56D		
	0%		17.70			25.20			37.35			41.15			25.41						4.02			5.20			6.91			8.54		
	15%		22.66			30.04			39.90			44.20			25.87						4.39			5.69			7.90			9.14		
	30%		25.90			32.40			43.94			50.39			6.16						5.21			7.14			9.57					
	45%		22.04			29.90			39.79			45.84			5.77						4.38			6.64			8.13			10.1		
	60%		18.13			26.83			35.14			42.65			5.19						4.20			5.77			7.91			9.86		

D—days, CPS—copper slag, S—sand.

## 5. Durability 

### 5.1. Water Absorption and Voids 

**Figure 15:**
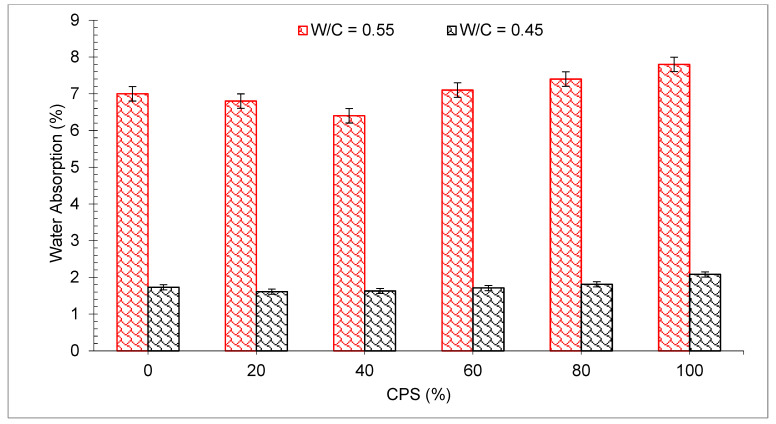
Water absorption of concrete: data source [47].

The average water absorption of the mix with CPS substation is shown in Figure 15. It can be noted that, except for the 20 percent CPS mix having *w*/*c* = 0.55, higher water absorption was exhibited compared with blank concrete. The *w*/*c* = 0.45 mixes showed less water absorption when compared to the corresponding control mix, except for the two mixes with the greatest CPS proportions, i.e., 80 and 100% which had the maximum water absorption. However, all the mixes had low water absorption rates of less than three percent. This was within predicted limits for high durability in terms of liquid infiltration in concrete, which was observed by the researcher [81]. The rise in water absorption, which was detected mostly in the *w*/*c* = 0.55 mixes, was caused by an excess of water, i.e., CPS grains showed less water absorption than sand, which might have resulted in a larger porosity of the mixture [47]. According to the findings of the research, up to 40% replacement of sand by CPS results in a general decrease in surface water absorption, after which the water absorption swiftly rises as the amount of CPS increases. The research indicates that the replacement of CPS for 40 percent of the cement resulted in a decrease in surface water absorption [61]. Similar trends were found with the rate of copper slag substitution, which increased up to 40% while the rate of surface water absorption reduced [82]. Water absorption of concrete with filler materials reduced because of the micro filling of spaces in the concrete, which led to a more compact mass and hence reduced the water absorption. However, due to the lack of flowability of filler materials, a larger dosage of filler materials might result in increased water absorption [7].

**Figure 16:**
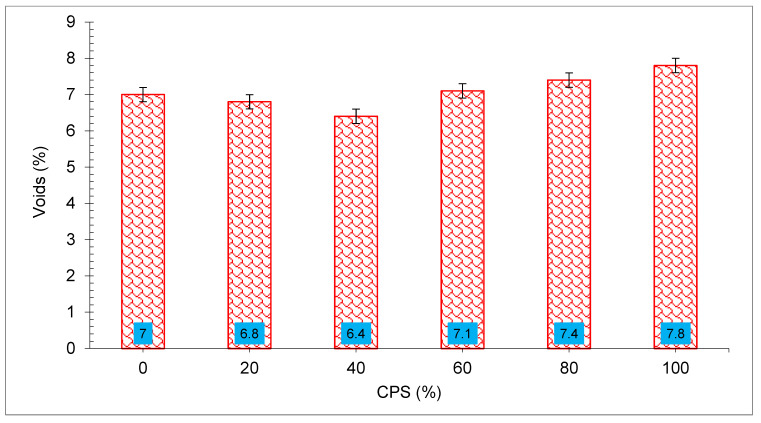
Voids in concrete: data source [33].

The percentage of voids in concrete mixes containing CPS is shown in Figure 16. It has been concluded that the voids contained in concrete mixtures follow a pattern similar to that of water absorption. The proportion of voids may decrease by 40% when fine aggregates are replaced with CPS. The percentage of voids in mix containing CPS 40% was determined to be 6.56 percent, which is the least void-containing of all the mixes. The presence of voids increased when CPS was substituted for more than 40% of the cement. Among all concrete mixes tested, the proportion of voids in CPS 100 percent concrete was the highest (7.82 percent). It was found that the 100% CPS mix had an even larger proportion of voids than the control mix. The settlement of CPS as a consequence of its heavy weight in contrast with natural causes water to rise to the surface, causing in cavities and a permeable microstructure on the surface [67]. The findings suggest that the substitution of 40% CPS as a partial alternative for natural sand will result in concrete that poses a comparable challenge to water absorption and cavities.

### 5.2. Accelerated Corrosion Testing Results

**Figure 17:**
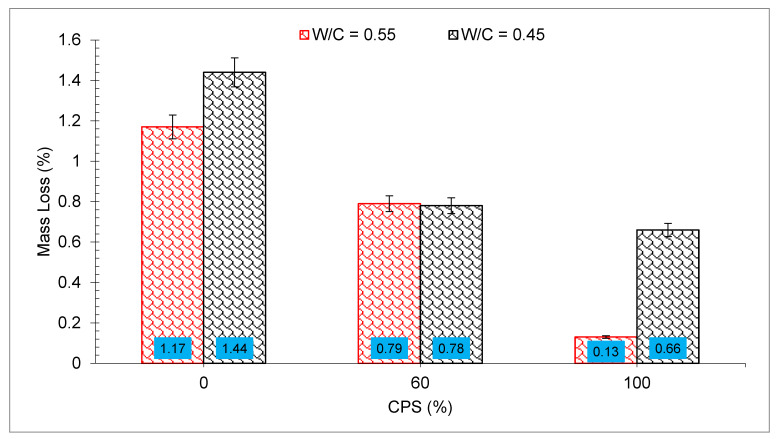
Corrosion test results: data source [47].

The results of the faster corrosion assessment in terms of mass failure of the entrenched reinforcement are depicted in Figure 17. Both control mixes (with *w*/*c* ratios of 0.45 and 0.55, respectively) showed more corrosion (as seen by the larger mass loss of the bars) than the concrete made with CPS. 

The 0.45 control mix had the greatest amount of corrosion, as well as several surface fractures near the reinforcing bar, which were the most severe. However, the *w*/*c* = 0.55 mix with 100 percent CPS demonstrated only a minor loss in mass compared to the other mixtures. Utilizing CPS as a substitute for sand, the researchers discovered that the corrosion resistance of the resultant mix was greater than that of mix made with natural sand. However, reverse results were examined by Brindha et al. [83], who demonstrated that for CPS utilized for up to 50% sand substitute concentrations, further raising CPS proportions resulted in a modest rise in the rust ratio when associated with blank concrete

### 5.3. Acid Resistance 

**Figure 18:**
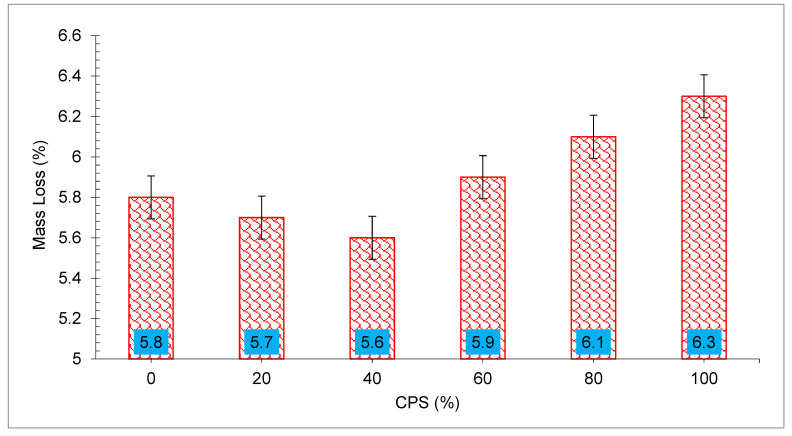
Acid resistance of concrete: data source [33].

The concrete including various amounts of CPS percentages was preserved in water for 28 days before being subjected to the sulfuric acid mixture for 56 days and examined for corrosion. The compressive capacity and mass of concrete sample were measured before and after contact with acid to determine the severity of the acid assault on the specimens. When exposed to a sulfuric acid solution for 56 days, the mass of concrete specimens varied, as shown in Figure 18. The results of the tests indicated that after 56 days of exposure to the sulfuric acid solution, all of the specimens lost weight. Weight loss could be minimized by up to 40% by increasing the percentage of CPS replacement. The CPS 40% mix had the smallest change in mass of all the mixes, with a change of just 5.63 percent. When comparing the drop in mass after 56 days of exposure for CPS 40% mix and the control mix of 0% CPS, it was found that the former was around four percent less. An excessive increase in the CPS fraction over 40% of sand has a negative effect on the ability to withstand the acid assault. There was a significant difference in mass between the 100% CPS concrete mix and the other mixtures tested. The change in mass for the 100% CPS concrete mix after 56 days of exposure was considerably larger than the change in the mass of the control mix.

### 5.4. Electrical Resistivity Results

**Figure 19:**
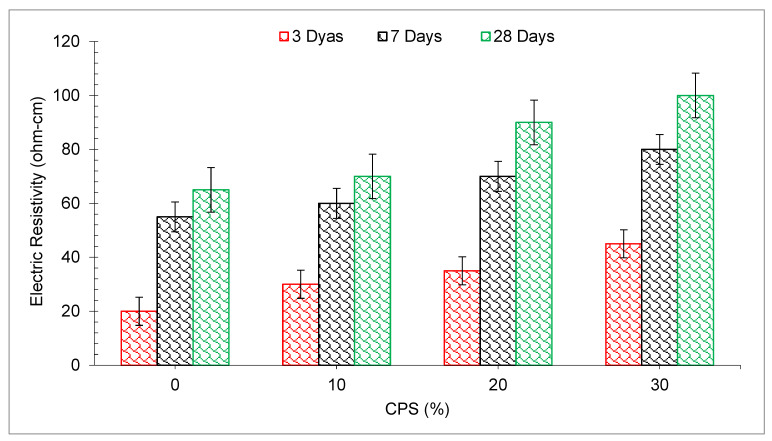
Electric resistivity of concrete: data source [64].

The electrical resistance is defined as the voltage age ratio multiplied by the electrical current that runs across a sample (voltage to current ratio). It is also referred to as material resistance to electric current in certain circles. Understanding the strength of the flow of the electrical current is important, since it may aid in predicting the probability of reinforcing corrosion occurring. The fluctuation in electrical resistance with hardening time and the percentage of CPS is shown in Figure 19. The electrical resistance of CPS cement mortars rose substantially over the duration of time. Resistance to 30% CPS mortars was much greater than that of 20% CPS mortars. The resistance of the control mortar did not grow with time, especially over a considerable length of time, and it remained lower than the resistance of the CPS mortars. The electrolytic current that passes through the wetting cement mortar is responsible for the wetness. The electrical resistivity may be used to determine porosity and permeability in a more indirect manner [84]. Mortar resistivity assessments are affected by a variety of parameters, including the makeup of the binder phase, the constitution of the liquid phase and the connectivity of the pore system [85].

### 5.5. Sulfate Resistance 

Extremely serious environmental deteriorations brought on by sulfate assault have an impact on the long-term durability of concrete buildings. Structures made of concrete, such as foundations, bridges, piers, concrete pipelines, etc., experience expansion and cracking as a result of sulfate assault, which worsens the condition. If the sulfate ions originate from sea water, ground water or soil, they will be present in the solution together with other ions including magnesium, sodium, calcium and potassium [86]. The findings demonstrate that adding CPS as an additional cementitious material to concrete increased its acidic resistance [87]. For improved resistance against concrete buildings vulnerable to sulfate assault, this research recommends using CPS as an alternative to fine aggregates with mineral admixtures [88].

In another investigation, iron slag was used as a replacement for fine aggregates in the SCC, and it was shown that specimens attacked by sulfate showed no mass loss despite developing white deposits after 91 days. After 28 days of sulfate exposure, it was discovered that each degree of iron slag replacement had a lower-than-5% effect on the compressive strength [89]. Sharma and Khan [67] stated that despite the improvement in the workability and compressive strength, the sulfate attack reduced as fine aggregate replaced up to 20% by CS. Najimi et al. [90] evaluated the durability of CS contained concrete exposed to sulfate attack. They claimed that use of CS as cement at 5%, 10% and 15%, caused deteriorative sulfate expansions decreased by 57.4%, 63.4% and 64.7%, respectively, compared with the control mixture. According to Gevaudan et al. [91] the addition of copper and cobalt to alkali-activated cement would lessen the rate of calcium sulfate generation and, as a consequence, would result in less permeability and corrosion when exposed to acidic environments. By forming a passivation and protective barrier against acid attack, copper and cobalt ions boost this cement’s acid resistance and decrease deterioration.

## 6. Scanning Electron Microscopy (SEM)

**Figure 20:**
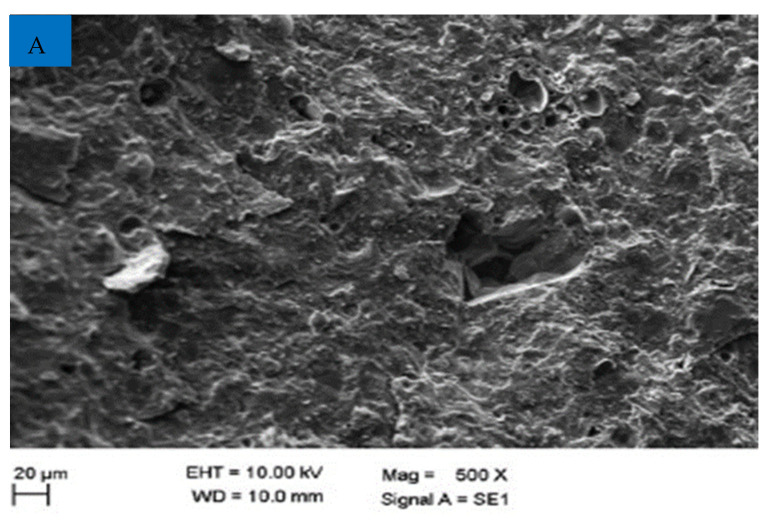

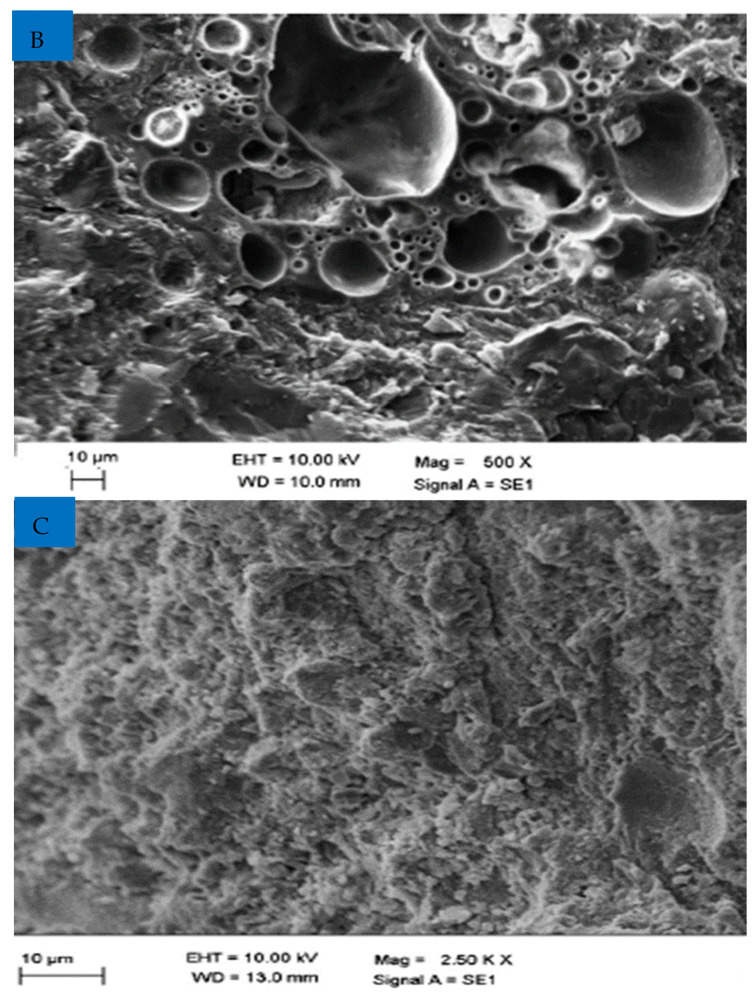
SEM Results, (**A**) 100% of CPS at 20 µm, (**B**) 100% of CPS at 10 µm and (**C**) 100% of CPS with 2% Nano Silica at 10 µm [74].

Figure 20A,B depict the SEM of concrete with full replacement of sand with CPS. It can be noted that the complete replacement of sand with CPS causes additional water to become stagnant in the concrete, resulting in an increase in the number of cavities and vessel channels in the finished product. The creation of these voids and capillary channels has an influence on the interlocking connection among the cement and the aggregates, causing in a loss of capacity, while the durability of the concrete will be affected because of the poor connection of cement paste with aggregate. However, the performance of concrete with 100% CPS can be improved with the supplement of secondary cementitious or filler materials. Combining pozzolanic reaction and filling voids of mineral admixture improved the performance of concrete [92,93]. Research was carried out using nanosilica with 100 percent CPS as fine aggregate in concrete [74]. Nano silica particles have a filler effect which causes an extremely dense structure to form because of their presence. The addition of nanosilica to concrete helps to prevent segregation and bleeding while also improving the cohesiveness of the concrete. A little amount of nanosilica is added to the cementitious matrix to lower its viscosity, offset the detrimental effects of trapped air and reduce the permeability of the cured concrete. From Figure 20C, it is noted that nanosilica (as pozzolanic material [94]) reacts with calcium hydroxide, and the reaction starts the creation of the secondary C-SH gel. This secondary C-S-H gel fills all of the pores in the solid state, making the concrete compact and improving its load capacity attributes.

## 7. X-Ray Diffraction (XRD) 

X-ray diffraction (XRD) patterns after 7, 28 and 90 days of curing and the blended cement pastes containing 30 percent copper slag are shown in Figure 4. To explore how the hydration products of blended cement vary with curing time, the spectra in the range of 2θ between 10 and 50 degrees are stacked. Similar groups of diffraction peaks may be seen in the mixed cements of various ages. It is obvious that the portlandite (CH), calcite (CaCO_3_), larnite (C_2_S), and ettringite are the major minerals in the paste samples [95].

Portlandite is produced when cement hydrates, and it is the main crystalline mineral in the pastes. A study also claimed that CH forms weak pockets which adversely affect the strength of concrete [93]. Furthermore, CH is chemically active, reacting other chemicals and resulting deterioration of the concrete structure [96]. The carbonation of CH during the setting and hardening of the pastes is to blame for the presence of calcite [97]. Due to its poor reactivity, C_2_S is present as a non-hydrated cement component [98]. In addition, it is challenging to use XRD to identify calcium silicate hydrates (C-S-H) gel, which is often classified as an amorphous phase. C-S-H gel improved the binding properties of cement paste, which results in more mechanical strength durability. 

**Figure 21:**
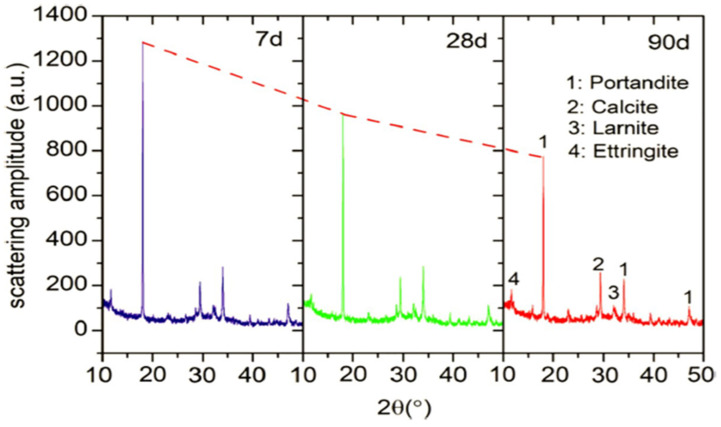
XRD of cement paste with 30% copper slag [99].

It is generally known that the reactive element in pozzolanic materials, amorphous silica, may interact with CH to create more hydration products. The consumption of CH in cement pastes is often used to measure the intensity of pozzolanic reaction. In mixed cement pastes, the CH diffraction peaks are located at 18.02 and 34.05 degree. As indicated in Figure 21, the peak CH intensity at 18.02 has been compared.

The findings indicate that this peak intensity gradually and noticeably decreased throughout the course of the curing process, with a residual amount still present in the pastes after 90 days. This suggests that the pozzolanic reaction results from the inclusion of UGCS and would continue even after 90 days of cure time. In the part that follows, a quantitative study will be carried out while taking the impact of CaCO_3_ into account to precisely quantify the level of pozzolanic reaction [97].

## 8. Hazards and Safety of Copper Slag 

Copper slags are used in the production of cement, aggregates, landfill, ballast, abrasives, roofing granules, glass, tiles and bituminous pavements, among other things. Copper slag has lately been studied in terms of its properties and applications [35]. The biggest worry in the large-scale usage of copper slags, however, is the fear of environmental danger due to heavy metal leaching from the slag and its long-term stability under harsh environmental conditions. The chemical compositions of the slags reveal extremely low heavy metal concentration, and the leach test findings demonstrate that simulated leachate in an aggressive laboratory test removes very little of any of these metals. The quantities eliminated are substantially below the regulated limits set by the US (and possibly other national) drinking water quality standards [100]. A similar study also claims that the leach test conservatively predicts that the slags will not release enough As, Cd, Cr, Pb, or Se to damage groundwater [100].

However, The Cu and Pb concentrations exceed the authorized limits (1.0 and 0.3 mg/L, respectively) at the 30 percent replacement level of CPS, whereas the other elements (Zn, Mn, Ni, Cr and As) are within the limits. Cr has the lowest concentration (it is close to the detection limit of 0.003 mg/L). When the CPS content is raised to 50 wt.%, the excess quantities of Cu and Pb concentrations rise as well compared with the 30 wt.% content instances. Excess concentrations of the metals Zn, Ni and As are also found. The findings clearly reveal that employing CPS (both air cooled and water cooled) as a partial cement replacement offers a significant danger of heavy metal leaching into the environment (namely, Cu, Pb, Zn, Ni and As), particularly at high replacement levels. Other CPS applications, such as aggregates in concrete and raw materials for alkali activation, may also provide a risk of heavy metal leaching [101].

The results of the leaching procedure (TCLP), acid leaching and repeated extraction tests performed on a large number of slag samples of varied compositions produced from the use of numerous copper concentrate show that heavy metals have little leachability and that long-term stability is assured even in harsh environments. Leaching experiments on mechanically activated samples provide an indication of the heavy metals’ resistance to leaching even after weathering. The heavy metals contained in the slag are stable, according to numerous extractions leaching experiments, and are unlikely to dissolve considerably even after repeated leaching in an acid rain environment. The greatest concentration of all components is significantly below the USEPA 40CFR Part 261 permitted limits [102]. A study [102] also observed that the heavy metals included in the slag are relatively stable and have low leachability, according to the TCLP, repeated extraction process tests and sulfuric acid leaching findings. The TCLP test results are far below the USEPA’s 40CFR Part 261 standards. The heavy metals found in the slag are very stable, according to several extraction leaching experiments, and are unlikely to dissolve considerably even in acid rain in a natural setting. The greatest concentration of elements recovered by the multiple extraction technique is less than the USEPA 40CFR Part 261 mandated limits for the elements covered by this standard.

It is indicated that the slag is safe for use in a broad range of applications, including Portland cement, building materials such as tiles and bituminous pavement projects. The slag samples are non-toxic and pose no risk to the environment.

## 9. Conclusions

The focus on green construction has resulted in an ongoing search for alternative materials to be employed in concrete construction. In this review, a complete parametric analysis was carried out to determine the impacts of CPS on physical and chemical properties, concrete qualities both fresh and hardened and concrete’s long-term durability performance. Consequently, the findings are given below.

- The physical property of CPS shows that the particle nature of CPS is rough and angular which adversely affects the flowability of concrete.
- The chemical composition of CPS ensures that it can be used as binding material.
- The slump value of concrete was reduced with the replacement of CPS due to angular and rough surface texture.
- The setting time increased with CPS as the pozzolanic reaction proceeded slowly.
- CPS up to 60% can be used without any harmful impact on the mechanical strength of concrete. The improvement in compressive, split tensile strength and flexure at 28 days was 9%, 6% and 9% higher than control concrete, respectively.
- The higher dose of CPS (80 and 90%) resulted in a decline in the mechanical strength of concrete due to the absence of flowability.
- A good correlation was observed between two specified strengths with an R^2^ value greater than 90%.
- The durability performance of concrete, such as water absorption and voids, corrosion resistance, acid resistance and electric resistivity increased with CPS.
- SEM results reveal that the performance of concrete with CPS can be improved with the addition of secondary cementitious materials.

The overall studies demonstrate that the CPS has the credibility to be utilized partially in concrete, either as a binding material or as a sand. The optimal percentages are an essential parameter for good strength. Different researchers recommend a different optimum value of CPS due to a change of source. The typical range of optimum value of CPS is from 50 to 60% by weight of fine aggregate. Furthermore, less information is available on dry shrinkage and creep properties of concrete with CPS. No or little information is available about the alkali silica reaction (ASR) connected with the CPS substitution. Lastly, although CPS can be utilized in concrete and the mechanical capacity can be enhanced, concrete is still low in tension. Therefore, further research was suggested to enhance the ductility of concrete with the supplement of various kinds of fibers. A study [103] concluded that the optimum mix substituted silica fume and copper slag for 7% and 20% of the cement, respectively, to provide a workable, resilient, cost-effective and durable mix design. However, there is less information about the economic benefits of CPS, and detailed investigation is required.

## Figures and Tables

**Table 1:** Physical Aspects of CPS.

Authors	Manjunatha et al. [45]	Jabri et al. [46]	Mavroulidou et al. [47]	Raju et al. [30]	Maharishi et al. [33]
Specific gravity	3.51	2.4	-	3.52	3.30
Water absorption (%)	0.36	-	0.11	-	0.36
Fineness modulus	3.11	-	2.97	3.68	3.18
Moisture content (%)	-	-	-	-	-
Density (kg/m^3^)	-	-	3.73	-	-
Specific surface area, (m^2^/kg)	-	-	-	-	-
Initial setting (min)	-	250	-	-	-
Fineness (cm^2^/g)	-	1261	-	-	-

**Table 2:** Chemical compounds of CPS.

Authors	Raju et al. [30]	Najimi et al. [53]	Jabri et al. [54]	Chithra et al. [55]	Rajasekar et al. [48]
SiO_2_	25.84	9.57	33.05	25.84	27
Al_2_O_3_	0.22	4.43	2.79	0.22	3.0
Fe_2_O_3_	68.29	57.42	53.45	68.29	0.60
MgO	-	1.56	1.56	-	4.0
CaO	0.15	22.5	6.06	0.15	63
Na_2_O	0.58	1.47	0.28	0.58	-
K_2_O	0.23	-	0.61	0.28	1.3

## Data Availability

All the data are available in manuscript.
